# Acute Hepatic Porphyria Should Be Included in the Diagnostic Work-Up of Patients with Resistant Hypertension or Suspected Secondary Hypertension

**DOI:** 10.3390/medsci13010014

**Published:** 2025-02-06

**Authors:** Paulo de Lima Serrano, Bruno de Mattos Lombardi Badia, João Paulo Barile, Patrícia Marques Mendes, Renan Brandão Rambaldi Cavalheiro, Kaliny Oliveira Peixoto, Igor Braga Farias, Roberta Ismael Lacerda Machado, Daniel Delgado Seneor, Wladimir Bocca Vieira de Rezende Pinto, Acary Souza Bulle Oliveira, Paulo Sgobbi

**Affiliations:** Porphyria Unit, Division of Neuromuscular Diseases, Federal University of São Paulo (UNIFESP), São Paulo 04039-060, SP, Brazil; pserrano@huhsp.org.br (P.d.L.S.);

**Keywords:** acute hepatic porphyria, hypertension, acute intermittent porphyria, hypertensive crisis, malignant hypertension, antihypertensive agents

## Abstract

Secondary hypertension and resistant hypertension may result from potentially treatable acquired or hereditary diseases. Inherited Metabolic Disorders are not routinely included in the differential diagnosis of these contexts associated with hypertension, despite the key importance of diagnosis for several of them which enable the early treatment of them. We aim to discuss the current evidence that indicates that a significant portion of cases of unknown resistant hypertension or suspected secondary hypertension may result from unrecognized Acute Hepatic Porphyria (AHP). Diagnostic work-up for AHP is not routinely performed during the evaluation of patients with resistant or refractory hypertension nor in the investigation of secondary hypertension. AHP may present both with neurological and systemic involvement, and hypertension may be observed as part of acute dysautonomia during acute neurovisceral attacks and as a chronic complication during disease course. As AHP represent a potentially treatable group of metabolic disorders, clinicians should consider the inclusion of this group in the diagnostic evaluation of patients with secondary or resistant hypertension.

## 1. Introduction: Concepts and Recommendations About Systemic Arterial Hypertension

Systemic arterial hypertension is a chronic condition common in the general population (estimated almost 31% of global prevalence) and poses a significant potential for chronic clinical complications and an increased risk of severe cardiovascular events, representing a central cause of cardiovascular morbidity and mortality [1]. Essential or primary hypertension is commonly considered in individuals with systolic blood pressure ≥ 130 mmHg and/or diastolic blood pressure ≥ 80 mmHg, with no specific etiological basis associated with the occurrence of hypertension [1]. There are, however, some variations of formal definitions of arterial hypertension, according to the different guidelines recently published: (i) 2024 European Society of Cardiology Guidelines [2]: defined by systolic blood pressure ≥ 140 mmHg or diastolic blood pressure ≥ 90 mmHg, in outpatient evaluation, and confirmed by out-of-office or one additional outpatient measurement–daytime ambulatory and home blood pressure monitoring assessments disclosing systolic blood pressure ≥ 135 mmHg or diastolic blood pressure ≥ 85 mmHg are also confirmatory; (ii) 2023 European Society of Hypertension [3]: defined by systolic blood pressure ≥ 140 mmHg or diastolic blood pressure ≥ 90 mmHg identified in repeated office assessments. Almost 50% of patients with essential hypertension have association with genetic mechanisms [4].

Secondary hypertension is defined by the occurrence of elevated blood pressure in individuals with identifiable underlying causes, which could be potentially treatable or significant for decision-making in the treatment of a patient. The most commonly included causes of secondary hypertension in diagnostic work-up in clinical practice are primary aldosteronism, renovascular disease, obstructive sleep apnea, chronic kidney disease, hypothyroidism, hyperthyroidism, Cushing’s syndrome, drug-induced hypertension, pheochromocytoma, paraganglioma, hyperparathyroidism, coarctation of the Aorta, pre-eclampsia, neurofibromatosis type 1, and gestational hypertension [1,5,6]. Secondary causes are generally considered in the differential diagnosis of patients with refractory and resistant hypertension, in patients with a known family history of young-onset hypertension, abrupt-onset hypertension, and cases with recognizable triggers of hypertension [1,5,6]. There is still, however, a significant number of cases in clinical practice where, despite the routine currently employed in investigating the classic etiologies of secondary hypertension, no specific etiological or etiopathogenic basis is established, leaving many of these cases as idiopathic or even misleading diagnosis of essential hypertension.

Resistant arterial hypertension is defined as any scenario in which blood pressure remains elevated above the target values planned in treatment, despite the use of three or more antihypertensive drugs at the most optimized and tolerated doses. The most commonly observed contexts of suspected resistance are related to other factors that lead to a false interpretation of elevated blood pressure (referred to as pseudo-resistance), such as improper blood pressure measurement, white coat effect-related hypertension, non-optimized therapeutic doses, or low adherence to treatment [7]. Refractory arterial hypertension, on the other hand, is characterized by persistently elevated and uncontrolled blood pressure despite optimized therapy, requiring the use of five or more antihypertensive drugs from different classes, typically including long-acting thiazide and spironolactone [7]. The occurrence of resistant or refractory hypertension usually necessitates an imperative investigation of secondary causes of arterial hypertension [5]. These scenarios are also more commonly associated with higher rates of complications, both directly and indirectly related to elevated blood pressure.

Malignant hypertension represents a clinical context of severe hypertensive emergencies with excessive elevation of blood pressure combined with the progression of accelerated disease, leading to microvascular damage in target organs, including retinal, renal, cardiac, and brain involvement, due to failure in autoregulatory mechanisms [8]. Nonadherence to antihypertensive drugs, drug-related hypertension (e.g., antiangiogenic and immunosuppressive agents), primary renal parenchymatous diseases, chronic kidney disease, and the other causes of resistant and secondary hypertension previously described [8]. As in cases of secondary hypertension, there is a significant number of patients with malignant hypertension in whom it is not possible to confirm a specific underlying etiology for the occurrence of this cardiovascular complication, leading to a significant increase in the risk of cardiovascular mortality.

## 2. Acute Hepatic Porphyria: Commonly Under-Recognized Inherited Metabolic Disorders with Both Acute and Chronic Complications

Acute Hepatic Porphyria (AHP) is a group of rare inherited metabolic disorders of the heme group biosynthesis pathway, which can be associated with neurological and systemic compromise [9,10]. Four different genetic conditions are classified as AHP, including Acute Intermittent Porphyria (AIP), Variegate Porphyria (VP), Hereditary Coproporphyria (HCP), and Doss porphyria or delta-aminolevulinic acid (ALA) dehydratase [9,11]. Pathophysiological mechanisms involve the accumulation of neurotoxic intermediates, such as ALA and porphobilinogen (PBG), and their direct and indirect effects in both the central and peripheral nervous system and in other tissues [12,13]. To a minor extent symptoms and signs may results partially from the absence of normal heme synthesis and its partial depletion, resulting in lack of products which require the heme group to their normal function and structure (e.g., cytochrome P450 isoenzymes, mitochondrial respiratory chain cytochromes, nitric oxide synthase, tryptophan dioxygenase, peroxidase, catalase, and other enzymes) [12,14].

Classically, AHP results in severe acute impairment triggered by decompensation factors, which lead to an increased demand on the heme biosynthetic pathway. This pathway has a functional deficit in one of the four enzymes associated with AHP [9,13]. Acute clinical presentations typically include high-intensity abdominal pain, Neuropsychiatric disturbances (notably behavioral disorders, sleep-wake cycle dysfunction, and acute encephalopathic changes), dysautonomia (including hypertension), and signs related to acute flaccid paralysis due to motor axonal polyneuropathy (usually severe and rapidly progressive) [9,11,12]. Central nervous system dysfunction may also occur in this context, such as posterior reversible encephalopathy syndrome (PRES), seizures, and status epilepticus, as well as photosensitivity-related skin lesions (in VP and HCP), metabolic disturbances such as Hyponatremia secondary to Syndrome of inappropriate antidiuretic hormone secretion (SIADH), and occasional changes in urine coloration [13,15]. It is not obligatory for all events related to the typical manifestations of acute neurovisceral crisis to occur in every episode of acute decompensation in patients with AHP to characterize an acute attack of this group of diseases. Early identification and management of milder forms of decompensation are ideal and recommended, aiming for a better clinical response to treatment measures [9,11].

Chronic manifestations related to AHP have been increasingly recognized, shifting the perspective on both the diagnosis and care of patients with AHP. Chronic neuropathic pain, chronic fatigue, and chronic psychiatry disturbances represent some of the most commonly observed scenarios. Additionally, other chronic clinical complications have become more identified and valued in recent decades, notably chronic kidney disease, systemic arterial hypertension, and an increased risk of hepatocellular carcinoma [16,17,18,19].

## 3. Cardiovascular Complications in AHP and Other Inherited Metabolic Disorders

Although systemic arterial hypertension is a recognized clinical manifestation and common complication in patients with various hereditary metabolic diseases (e.g., Fabry’s disease and homocystinuria), it is usually less addressed as an isolated cardiovascular condition compared to other cardiovascular and cardiac manifestations, such as cardiomyopathies, cardiac arrhythmias, and valvular heart disease [20]. The impact that certain metabolites and inherited metabolic disorders may have on the endothelium and myocardium remains largely unknown, despite these aspects being somewhat better understood in relation to the endothelium and renal dysfunction in AHP [21,22].

Several distinct cardiovascular complications have been recognized in association with AHP, including acute myocardial infarction, arterial hypertension, cardiac tachyarrhythmias, acute dysautonomia, hypertensive emergencies, and cardiomyopathies, including Takotsubo syndrome [19,23,24,25,26,27,28,29,30]. Both delayed AHP diagnosis and the unavailability of specific AHP treatments lead to higher rates of complications and mortality rates between 5% and 20% [31]. The AIPRACUS (Acute Intermittent Porphyria Related Abnormalities in Cardiovascular System) study, coordinated by the National Institute of Cardiology and Institute of Hematology and Transfusion Medicine, in Warsaw, Poland (NCT05882136), is prospectively characterizing a large cohort of patients with AIP who have experienced at least one hospitalization due to acute exacerbation. The study aims to investigate cardiac morphology, biomarkers of myocardial injury and heart failure, blood pressure-related aspects, electrocardiographic patterns, cardiac arrhythmias, and cardiovascular risk factors in the studied population [31]. Data from the AIPRACUS study will still be published later.

It is believed that systemic arterial hypertension in the context of AHP results from a complex range of multiple factors, including neurotoxic metabolites (mainly ALA), autonomic dysregulation (due to the acute and chronic dysautonomic effects of the disease), secondary toxic endothelial dysfunction, and the chronic effect of alteration of renal metabolic function induced by AHP [21,22,31]. Renal dysfunction may result also from mechanisms related to mitochondrial oxidative lesions induced by ALA, chronic tubulointerstitial lesions, and toxic aggregation of porphyrins in the proximal tubule [22].

A high prevalence of both systemic arterial hypertension and chronic kidney disease has been estimated based on real-world studies in patients with AHP. The prevalence of arterial hypertension ranges from 30% to 75% in most populations with AHP [19,31,32] and around 35.1% in the Brazilian population with AHP [33]. In various contexts within the current medical literature, case series and individual case reports have highlighted the correlation between systemic arterial hypertension and AHP, particularly in conjunction with other typical signs and symptoms of this group of disease during acute decompensation, as well as in cases without typical signs. Reports in this scenario include descriptions of hypertension as a chronic complication—such as in cases of hypertension in young individuals, secondary hypertension, and refractory hypertension—and as an acute complication, including hypertensive emergencies and accelerated malignant hypertension [32,34,35,36]. It is therefore quite surprising that none of the most important guidelines, position statements and recommendations include the possibility of AHP as a potentially treatable cause of secondary hypertensive, as well as do not mention the need of laboratory assessment for suspected AHP [2,3,37].

The importance of diagnosing AHP in association with cases of arterial hypertension lies in its implications for the risk of acute and chronic neurological and neuropsychiatric complications linked to AHP, as well as other chronic systemic complications, such as hepatocellular carcinoma and chronic kidney disease [25,38,39,40]. The diagnosis of AHP is also critically important to ensure the use of drugs with lower porphyrinogenic potential and greater safety in the treatment of arterial hypertension, as several prescription guidelines and manuals are available [14,34,41,42,43,44,45]. Therefore, in also confirmed cases of AHP or in patients with a high diagnostic suspicion for AHP presenting with arterial hypertension, it is recommended to routinely verify the safety profile of the drugs used in clinical practice in at least one of the several available and periodically updated databases [46,47,48]. According to recommendations from The Porphyria Information Centre at the University of Cape Town (available at: https://porphyria.uct.ac.za/porphyria-professionals/porphyria-professionals/prescribing-porphyria-treatment-specific-disorders-poprhyria/treatment-hypertension-heart-failure-porphyria (accessed on 10 January 2025)) [49], some important aspects may be considered when considering therapy choice: (i) beta blocking agents are generally considered safe for patients with AHP; (ii) Angiotensin II receptor blockers can be considered as possible therapies and generally safe, especially candesartan, valsartan and eprosartan; (iii) angiotensin converting enzyme inhibitors are considered safe as a group; (iv) calcium channel blockers have variable profile of risk, according to each drug, and generally should be limited to use with extreme caution, as there are other antihypertensive classes with much more safe profiles in patients with AHP; (v) most diuretics are considered safe, however it is importance to make a case-by-case decision, based on the profile of each drug and considering also experimental evidence of porphyrinogenicity, as there are other drug classes with better safety profiles; (vi) methyldopa, hydralazine and dihydralazine should be avoided due to their porphyrinogenic evidence. A summary of the porphyrinogenicity and safety profiles of the main antihypertensive drugs used in clinical practice is showed in Table 1, according to the Acute Porphyria Drug Database/NAPOS (available online: https://drugsporphyria.net/clear/1 (accessed on 10 January 2025)) [47].

## 4. Conclusions

We highly recommend clinicians to consider the inclusion of AHP within the routine diagnostic work-up of causes of secondary systemic arterial hypertension, regardless of the existence of a known family history or classic signs of systemic involvement of AHP. AHP represents underdiagnosed but potentially treatable inherited metabolic disorders for which early and adequate diagnosis offers the possibility of adopting measures to prevent chronic complications and severe acute metabolic decompensation. The diagnosis of AHP additionally enables the investigation and tracking of family members who may be affected. Knowledge regarding the choice of drugs with a lower porphyrinogenic risk is essential in considering the best therapeutic options in the context of treating systemic arterial hypertension, therefore it is essential to consider the differential diagnosis of AHP in cases of secondary hypertension, refractory hypertension and malignant hypertension. None of the currently published recommendations and guidelines on systemic arterial hypertension [2,3,37] have included AHP investigation during the diagnostic work-up of patients with resistant or refractory arterial hypertension. We also recommend that future guidelines and clinical directives on arterial hypertension consider the inclusion of AHP in the differential diagnosis of secondary hypertension. This would enable a greater number of clinicians and healthcare institutions to incorporate the necessary diagnostic evaluation for AHP into routine clinical practice and increase disease awareness.

## Figures and Tables

**Table 1:** Safety profiles of some of the drugs used in the treatment of systemic arterial hypertension in patients with AHP, according to the Acute Porphyria Drug Database/NAPOS (accessed on 10 January 2025) [47]. Data about the safety of combined therapies from different antihypertensive drug classes may be also checked in the Acute Porphyria Drug Database website. The safety profile summarized in this table for single isolated molecules does not necessarily reflect the safety of combined presentations with other drugs. Legend: CYP: cytochrome P450.

Antihypertensive Therapy	Porphyrinogenicity/Safety
**Non-selective beta blocking agents**	
Propranolol	Not porphyrinogenic.
Alprenolol	Unknown—prefer to avoid.
Sotalol	Not porphyrinogenic.
Nadolol	Unknown—prefer to avoid.
Pindolol	Probably not porphyrinogenic.
**Selective beta blocking agents**	
Metoprolol	Not porphyrinogenic.
Atenolol	Not porphyrinogenic.
Bisoprolol	Probably not porphyrinogenic.
Esmolol	Not porphyrinogenic.
Nebivolol	Unknown—prefer to avoid.
**Alpha and beta blocking agents**	
Labetalol	Not porphyrinogenic.
Carvedilol	Probably not porphyrinogenic.
**Calcium channel blockers—Dihydropyridine derivative**	
Amlodipine	Probably not porphyrinogenic.
Felodipine	Probably not porphyrinogenic.
Isradipine	Probably not porphyrinogenic.
Nifedipine	Probably not porphyrinogenic.
Nicardipine	Unknown—prefer to avoid.
Nimodipine	Probably not porphyrinogenic.
**Calcium channel blockers—Phenylalkylamine derivatives**	
Verapamil	Probably porphyrinogenic. Potent CYP3A4 inhibitor.
**Calcium channel blockers—Benzothiazepine derivatives**	
Diltiazem	Probably porphyrinogenic. Potent CYP3A4 inhibitor.
**Angiotensin converting enzyme inhibitors**	
Captopril	Probably not porphyrinogenic.
Enalapril	Not porphyrinogenic.
Lisinopril	Not porphyrinogenic.
Perindopril	Probably not porphyrinogenic.
Ramipril	Probably not porphyrinogenic.
Quinapril	Not porphyrinogenic.
Benazepril	Unknown—prefer to avoid.
Cilazapril	Probably not porphyrinogenic.
Fosinopril	Probably not porphyrinogenic.
Trandolapril	Probably not porphyrinogenic.
Delapril	Unknown—prefer to avoid.
Imidapril	Unknown—prefer to avoid.
**Angiotensin II receptor blockers**	
Losartan	Probably not porphyrinogenic.
Eprosartan	Not porphyrinogenic.
Valsartan	Not porphyrinogenic.
Irbesartan	Probably not porphyrinogenic.
Tasosartan	Unknown—prefer to avoid.
Candesartan	Not porphyrinogenic.
Telmisartan	Probably not porphyrinogenic.
Olmesartan	Unknown—prefer to avoid.
Azilsartan medoxomil	Unknown—prefer to avoid.
**Renin inhibitors**	
Aliskiren	Probably not porphyrinogenic.
Remikiren	Unknown—prefer to avoid.
**Thiazide derivatives**	
Diazoxide	Unknown—prefer to avoid.
**Hydrazinophtalazine derivatives**	
Hydralazine	Porphyrinogenic. CYP1A2 inducer.
Dihydralazine	Porphyrinogenic. CYP1A2 inducer.
**Pyrimidine derivatives**	
Minoxidil	Unknown—prefer to avoid.
**Nitroferricyanide derivatives**	
Nitroprusside	Unknown—prefer to avoid.
**Diuretics—Thiazides**	
Hydrochlorothiazide	Probably not porphyrinogenic.
Bendroflumethiazide	Probably not porphyrinogenic.
Hydroflumethiazide	Unknown—prefer to avoid.
Methyclothiazide	Possibly porphyrinogenic.
**Diuretics—Sulfonamides**	
Chlortalidone	Unknown—prefer to avoid.
Clopamide	Unknown—prefer to avoid.
Indapamide	Unknown—prefer to avoid.
Furosemide	Probably not porphyrinogenic.
Bumetanide	Probably not porphyrinogenic.
Piretanide	Unknown—prefer to avoid.
**Aryloxyacetic acid derivatives**	
Etacrynic acid	Probably not porphyrinogenic.
**Diuretics—Aldosterone antagonist**	
Spirinolactone	Porphyrinogenic.
Eplerenone	Probably not porphyrinogenic.
Canrenoate	Possibly porphyrinogenic.
Finerenone	Unknown—prefer to avoid.
**Diuretics—Other potassium-sparing drugs**	
Amiloride	Not porphyrinogenic.
Triamterene	Unknown—prefer to avoid.
**Other (Miscellaneous)**	
Methyldopa	Porphyrinogenic.
Guanfacine	Unknown—prefer to avoid.
Reserpine	Unknown—prefer to avoid.
Doxasozin	Probably not porphyrinogenic.

## Abbreviations

The following abbreviations are used in this manuscript:

AHP	Acute Hepatic Porphyria
AIP	Acute Intermittent Porphyria
ALA	Delta-Aminolevulinic Acid
ALAD	Delta-Aminolevulinic Acid dehydratase
APF	American Porphyria Foundation
CYP	Cytochrome P450
HCP	Hereditary Coproporphyria
ICU	Intensive Care Unit
NAPOS	The Norwegian Porphyria Centre
NAPS	National Acute Porphyria Service
PBG	Porphobilinogen
PRES	Posterior reversible encephalopathy syndrome
UKPMIS	United Kingdom Porphyria Medicines Information Service
VP	Variegate Porphyria
